# Synthesis and characterization of low-molecular-weight π-conjugated polymers covered by persilylated β-cyclodextrin

**DOI:** 10.3762/bjoc.8.170

**Published:** 2012-09-11

**Authors:** Aurica Farcas, Ana-Maria Resmerita, Andreea Stefanache, Mihaela Balan, Valeria Harabagiu

**Affiliations:** 1 Inorganic Polymers, ‘‘Petru Poni’’ Institute of Macromolecular Chemistry, Grigore Ghica Voda Alley, 700487-Iasi, Romania

**Keywords:** alternating fluorene-bithiophene copolymer, cyclodextrins, interlocked molecules, macrocycles, persilylated β-cyclodextrin, polyrotaxanes

## Abstract

The paper reports the preparation of a poly[2,7-(9,9-dioctylfluorene)-*alt*-5,5'-bithiophene/PS-βCD] (**PDOF-BTc**) polyrotaxane copolymer, through a Suzuki coupling reaction between the 5,5^'^-dibromo-2,2'-bithiophene (**BT**) inclusion complex with persilylated β-cyclodextrin (PS-βCD), and 9,9-dioctylfluorene-2,7-bis(trimethylene borate) (**DOF**) as the blocking group. The chemical structure and the thermal and morphological properties of the resulting polyrotaxane were investigated by using NMR and FT-IR spectroscopy, TGA, DSC and AFM analysis. The encapsulation of **BT** inside the PS-βCD cavity results in improvements in the solubility, as well as in different surface morphology and thermal properties of the **PDOF-BTc** rotaxane copolymer compared to its noncomplexed **PDOF-BT** homologue. In contrast, the number-average molecular weight (*M*_n_) of **PDOF-BTc** rotaxane copolymer indicated lower values suggesting that the condensation reaction is subjected to steric effects of the bulkier silylated groups, affecting the ability of the diborate groups from the **DOF** molecule to partially penetrate the PS-βCD cavity.

## Introduction

Organic materials with extended π-conjugation have attracted considerable attention in recent years as a new class of active organic materials for optoelectronic applications [1–4]. Among the conjugated polymers, a number of poly(9-alkylfluorene)s (**PF**s) and poly(9,9-dioctylfluorene) (**PDOF**) polymers in particular have been the focus of much research as encouraging candidates for organic light-emitting diodes (OLEDs) due to their pure blue luminescence and high efficiency [5–10]. The application of **PF**s and **PDOF** conjugated polymers is limited by unwanted side effects, such as aggregation and a wide emission band during operation [8]. Taking into account the relevant photophysical properties of these organic compounds, new synthetic approaches were developed. Copolymerization of fluorene with thiophene, bithiophene or other aromatic comonomers is an alternative method for tuning the optical, electronic and thermal properties [11–18]. However, as a consequence of intramolecular interactions, these synthetic methods are often accompanied by undesirable side effects influencing the optoelectronic properties, e.g., red shifting or lower fluorescence.

As a route to candidate materials for use in molecular devices, the construction of mechanically interlocked molecules, such as rotaxanes and polyrotaxanes, has attracted considerable attention [19–23]. A rotaxane assembly comprises a macrocyclic component (host) encircling an axle (guest) through noncovalent interactions; bulky groups (also known as stoppers) are attached at the ends of the axle to prevent dethreading of the host.

In the past few years many authors have demonstrated that the encapsulation of conjugated polymers into macrocycle cavities plays an important role in the construction of diverse polymeric architectures. Moreover, the fabrication of mechanically interlocked molecules, such as polyrotaxanes, has been investigated as a method for the further improvement of thermal and electro-optical properties through the insulating backbones of conjugated polymers [20–24]. The studies revealed an attractive approach to achieve a higher degree of control over molecular rigidity, prevention of aggregation, fluorescence efficiency, improved solubility, and surface-morphological properties of the resulting conjugated polyrotaxanes [5,11,21,23,25–31].

Among the several known host molecules, e.g., crown ethers [32], cyclodextrins (CDs) have been employed for the synthesis of polyrotaxanes with π-conjugated polymers. As a result, many CD-based rotaxanes and polyrotaxanes have been reported until now [5,11–12,19–31]. In spite of the extended use of new compounds as hosts for inclusion-complex formation, CD derivatives have received less attention when compared to native CDs. Very few polyrotaxanes within CD derivatives were reported [12,33–34]. Recently, we have exemplified such improvements on the photophysical properties of **PF**s by using persilylated γ-CD as a new host molecule [33]. Inclusion of bithiophene into persilylated β-cyclodextrin, randomly methylated β-cyclodextrin, or chemically modified CD derivatives, followed by copolymerization with fluorene monomers results in significant changes in the thermal as well as photophysical stability, and the ability to form good films [12,34].

**Scheme 1:**
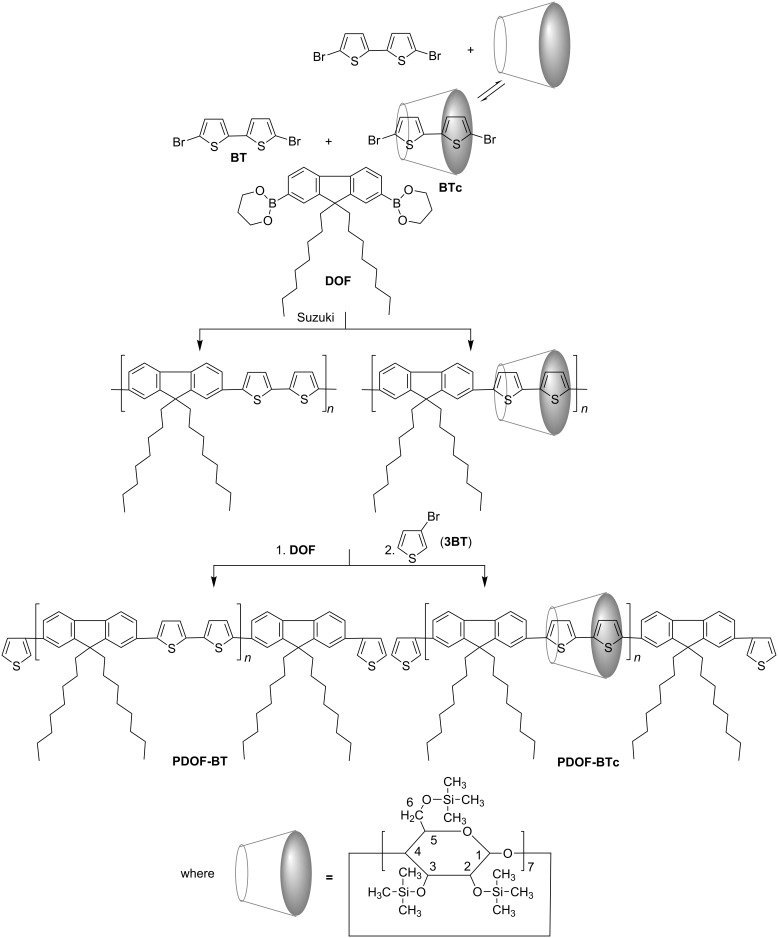
Synthesis of **PDOF-BT** and **PDOF-BTc** copolymers.

Herein, we report the preparation and characterization of a main-chain polyrotaxane with alternating fluorene–bithiophene moieties covered by persilylated β-cyclodextrin (PS-βCD). Thus, poly[2,7-(9,9-dioctylfluorene)-*alt*-5,5'-bithiophene/PS-βCD] (**PDOF-BTc**), was synthesized by Suzuki cross-coupling reaction between 5,5'-dibromo-2,2'-bithiophene (**BT**), as an inclusion complex in PS-βCD cavities, and the bulky molecule 9,9-dioctylfluorene-2,7-bis(trimethylene borate) (**DOF**), (Scheme 1). Of particular interest is the ability of cross-coupling reaction of **BT** and **DOF** to proceed close to the cavity surface of PS-βCD, which will provide a deeper insight into the use of these new host molecules (more soluble in nonpolar organic solvents) for the construction of mechanically interlocked molecules with conjugated polymers.

## Results and Discussion

The paper describes the preparation of poly[2,7-(9,9-dioctylfluorene)-*alt*-5,5'-bithiophene/PS-βCD] (**PDOF-BTc**) main-chain polyrotaxane and its noncomplexed **PDOF-BT** counterpart copolymer by Suzuki cross-coupling reaction. The preparation of copolymer polyrotaxane (**PDOF-BTc**) involves a 1:1 5,5^'^-dibromo-2,2'-bithiophene inclusion complex in PS-βCD (**BTc**) and 9,9-dioctylfluorene-2,7-bis(trimethylene borate) (**DOF**) as a bulky molecule followed by a small excess of **DOF** at the end of polymerization and, finally, 3-bromothiophene (**3T**) as a monofunctional end-capping reagent, as illustrated in Scheme 1. In order to analyse the influence of end-capping reagents on the photophysical properties we changed the termination of the growing chains to **3T** instead of bromobenzene, as in previously reported results [12]. A noncomplexed **PDOF-BT** copolymer was also synthesized by polycondensation reaction between **DOF** and **BT**, and its properties were compared with the rotaxane **PDOF-BTc** copolymer.

**Figure 1:**
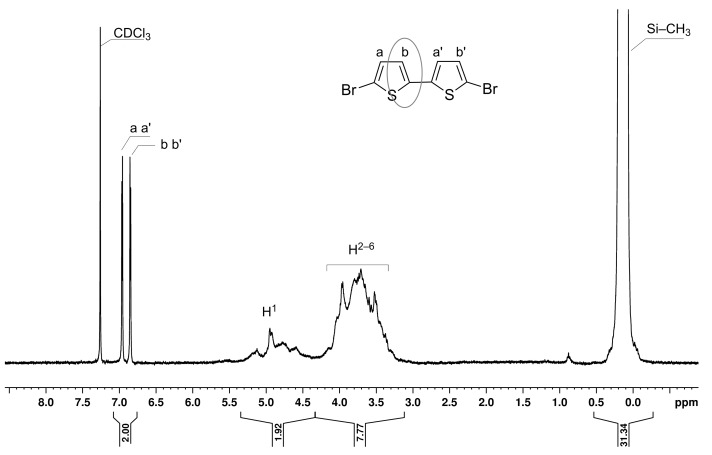
^1^H NMR spectrum (CDCl_3_) of the **BTc** inclusion complex.

The first step in the preparation of **PDOF-BTc** polyrotaxane is the threading of the **BT** monomer through the PS-βCD cavity to form the **BTc** inclusion complex. **BTc** obtained as a precipitate from a 2:1 mol/mol mixture of PS-βCD and **BT** in acetone was isolated, purified and characterized by ^1^H NMR, as can be seen in Figure 1. The average number of PS-βCD macrocycles per **BT** unit was calculated by using the ratio of the integrated area of the peak assigned to the H from –CH_3_ groups of PS-βCD (0.09–0.11 ppm, I_H-CH3_) and the proton peaks of **BT** (6.85–7.97 ppm, I_BT_), Figure 1. The average number of PS-βCD macrocycles per **BT** unit was calculated as (I_BT_/4)/(I_H-CH3_/63) and found to be 0.95 (i.e., ca. 95% coverage).

Not surprisingly, due to the presence of PS-βCD the rotaxane **PDOF-BTc** copolymer showed a marked contrast compared with **PDOF-BT**. The **PDOF-BTc** rotaxane copolymer was soluble (~10% by weight) in petroleum ether (after vortex stirring at room temperature for 15 minutes). The ^1^H NMR spectrum of a soluble fraction in petroleum ether indicated a higher coverage with PS-βCD. From this spectrum a PS-βCD/**BT** molar ratio of about 1/1.5 was determined, as can be seen in Figure S1 in the Supporting Information File 1.

**Figure 2:**
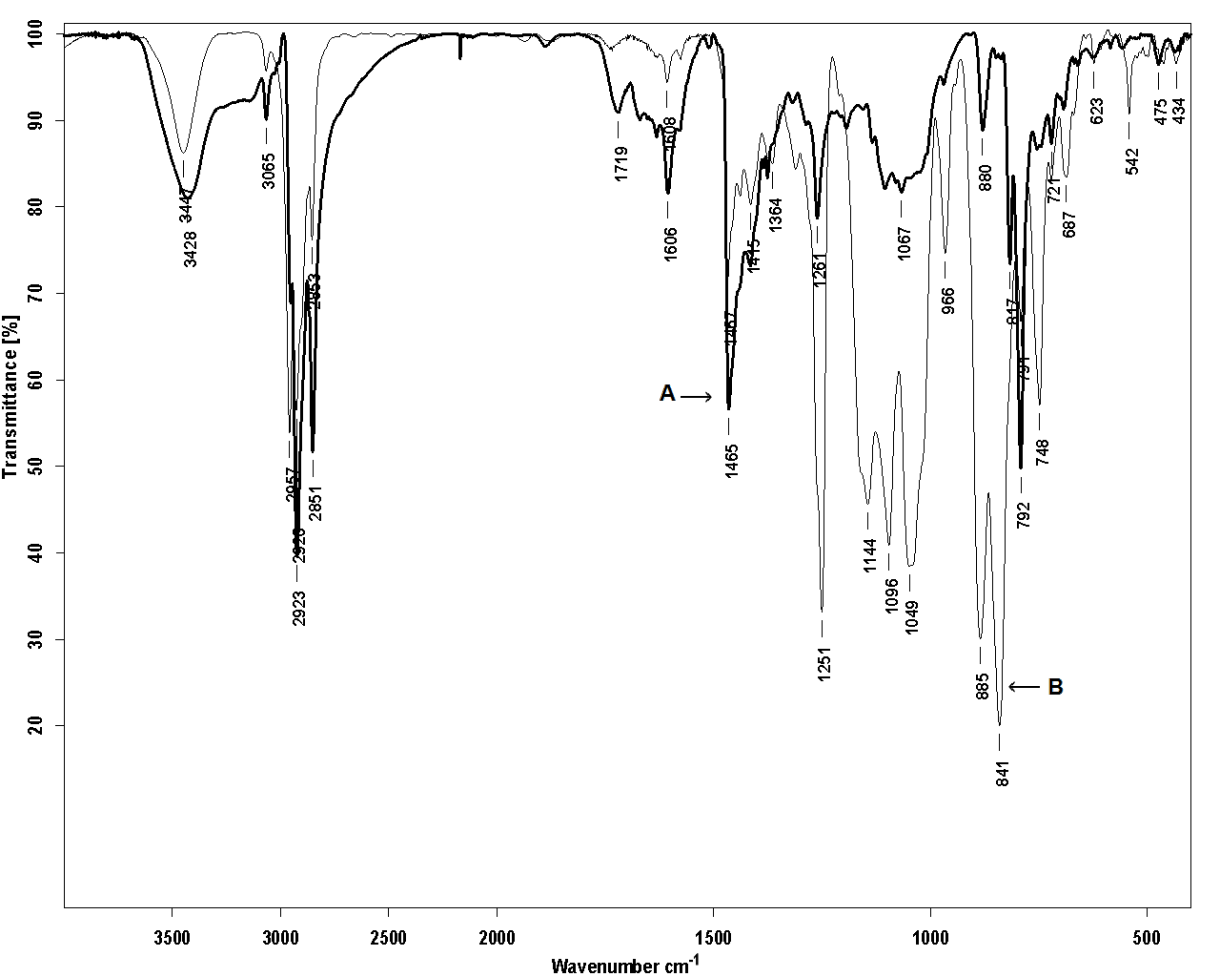
FTIR spectra (KBr pellet) of **PDOF-BT** (A) and **PDOF-BTc** (B) copolymers.

The chemical structure of **PDOF-BTc** (insoluble part in petroleum ether) and **PDOF-BT** copolymers was proved by ^1^H NMR and FTIR analysis. The infrared spectrum of the **PDOF-BTc** (Figure 2B) shows all the characteristic bands of **PDOF-BT** and additional bands located in the 748–1251 cm^−1^ region. Note that the peaks at 792 and 817 cm^−1^, assigned to the C–H out-of-plane bending vibrations, and the peak at 880 cm^−1^, assigned to the C–H in-plane vibrations of the aromatic rings, were at distinctly lower energy in **PDOF-BTc** as compared to the corresponding peaks of the non-rotaxane **PDOF-BT** copolymer.

**Figure 3:**
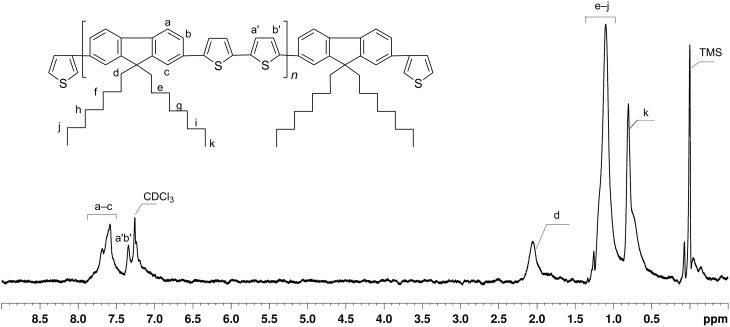
^1^H NMR spectrum of **PDOF-BT** copolymer (CDCl_3_).

**Figure 4:**
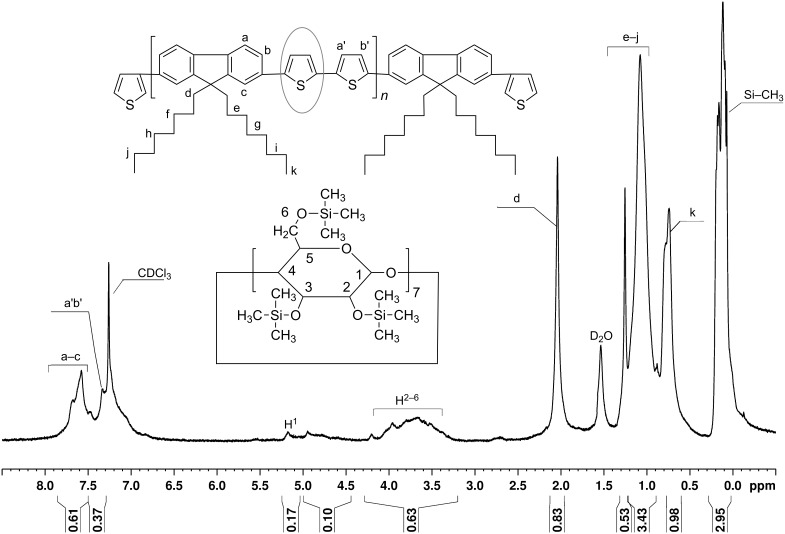
^1^H NMR spectrum of **PDOF-BTc** copolymer (CDCl_3_).

^1^H NMR spectra of **PDOF-BT** and **PDOF-BTc** (insoluble part in petroleum ether) are presented in Figure 3 and Figure 4, respectively. The NMR spectrum of **PDOF-BT** shows characteristic peaks for both **DOF** and **BT** chains in good agreement with the proposed structures.

The coverage of the rotaxane copolymer with the macrocycle, i.e., the average number of PS-βCD macrocycles per repeating unit [23], was determined from the NMR spectral analysis and was calculated from the ratio of the integrated area of the peak assigned to the H from –CH_3_ groups of PS-βCD (0.121–0.249 ppm, I_H-CH3_) and the proton peaks of **BT** (7.247–7.412 ppm, I_BT_), as shown in Figure 4. The average number of PS-βCD macrocycles per repeat unit was calculated as (I_BT_ /4)/(I_H-CH3_/63) and found to be 0.32 (i.e., ca. 32% coverage). The peaks corresponding to the fluorene copolymer chains in **PDOF-BTc** were down-field shifted by 0.03–0.08 ppm, as compared to the noncomplexed **PDOF-BT** sample (Figure 4).

The weight-average molecular weight (*M*_w_) and number-average molecular weight (*M*_n_), with the polydispersity index PDI (PDI = *M*_w_/*M*_n_), of **PDOF-BTc** and **PDOF-BT** samples were determined by gel-permeation chromatography (GPC) analysis, using polystyrenes as standards and CH_2_Cl_2_ as eluent. GPC data are listed in Table 1 and shown in Figure S2 and Figure S3 in the Supporting Information File 1. On the basis of the molecular weight, the numbers of repeat units for **PDOF-BTc** and **PDOF-BT** samples are 10 and 40, respectively. **PDOF-BT** non-rotaxane copolymer has higher molecular weights than the **PDOF-BTc** sample, an expected result owing to the lower accessibility of diborate to bromine groups encapsulated in the PS-βCD cavity. The higher PDI values of the **PDOF-BTc** copolymer compared to **PDOF-BT** can be attributed to variations in the average number of PS-βCD units per macromolecular chain (see incomplete coverage determined by ^1^H NMR above). It is important to note that the lower molecular weights of **PDOF-BTc** rotaxane copolymer, which have not been observed for other polyrotaxanes [5,29,31,34], can be assigned to the contribution from the structure of the PS-βCD macrocycle, which provides a deeper insight into the blocking effect of silylated groups on the cross-coupling reaction. In order to obtain higher-molecular-weight polyrotaxanes the optimal reaction time has to be taken into consideration.

**Table 1:** The molecular weights of the polymers.

Polymer	*M*_n_	*M*_w_	*M*_w_/*M*_n_

**PDOF-BTc**	14771	24805	1.67
**PDOF-BT**	23286	34461	1.48

**Figure 5:**
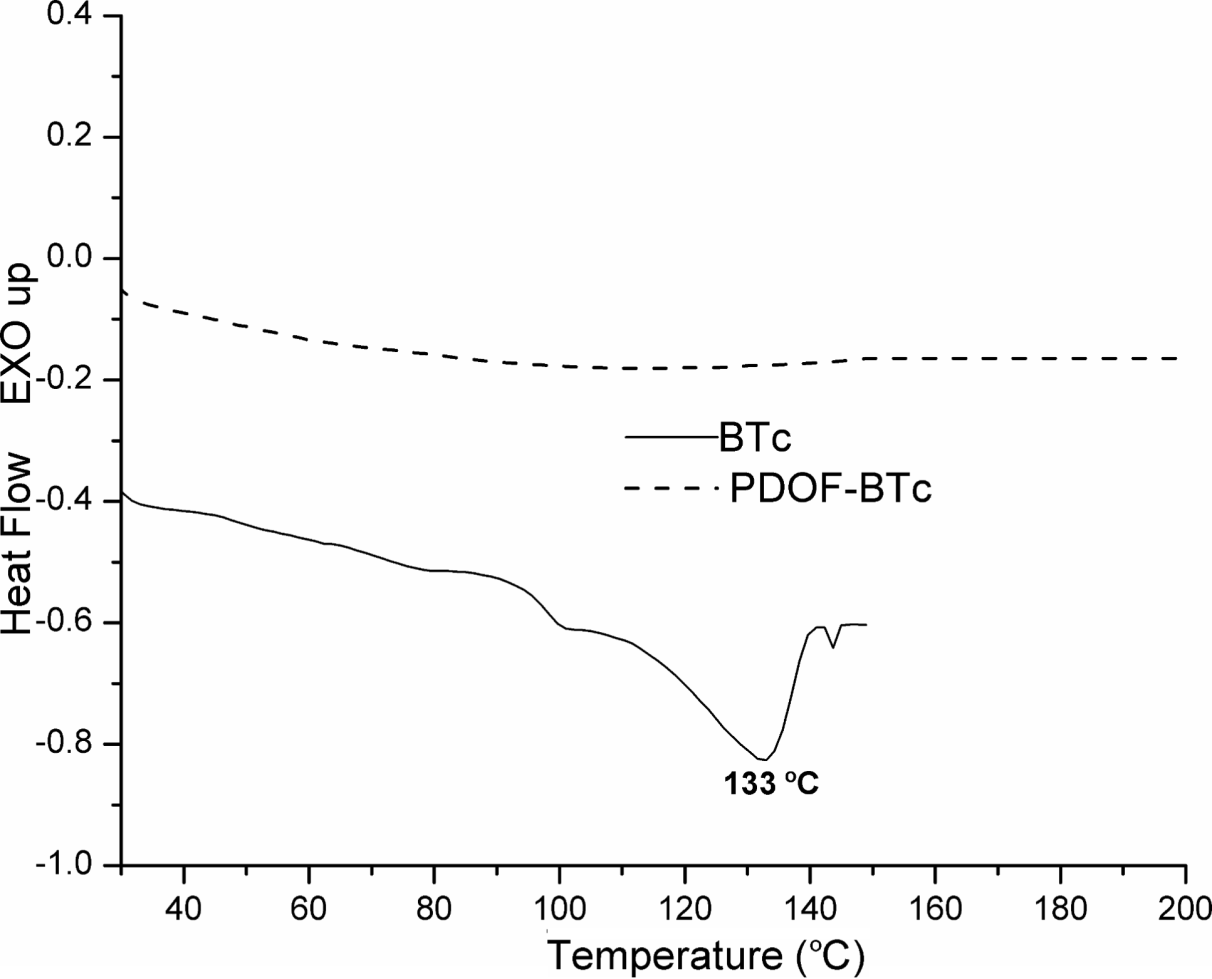
DSC curves of **BTc** and **PDOF-BTc** from second-heating DSC measurements.

**Figure 6:**
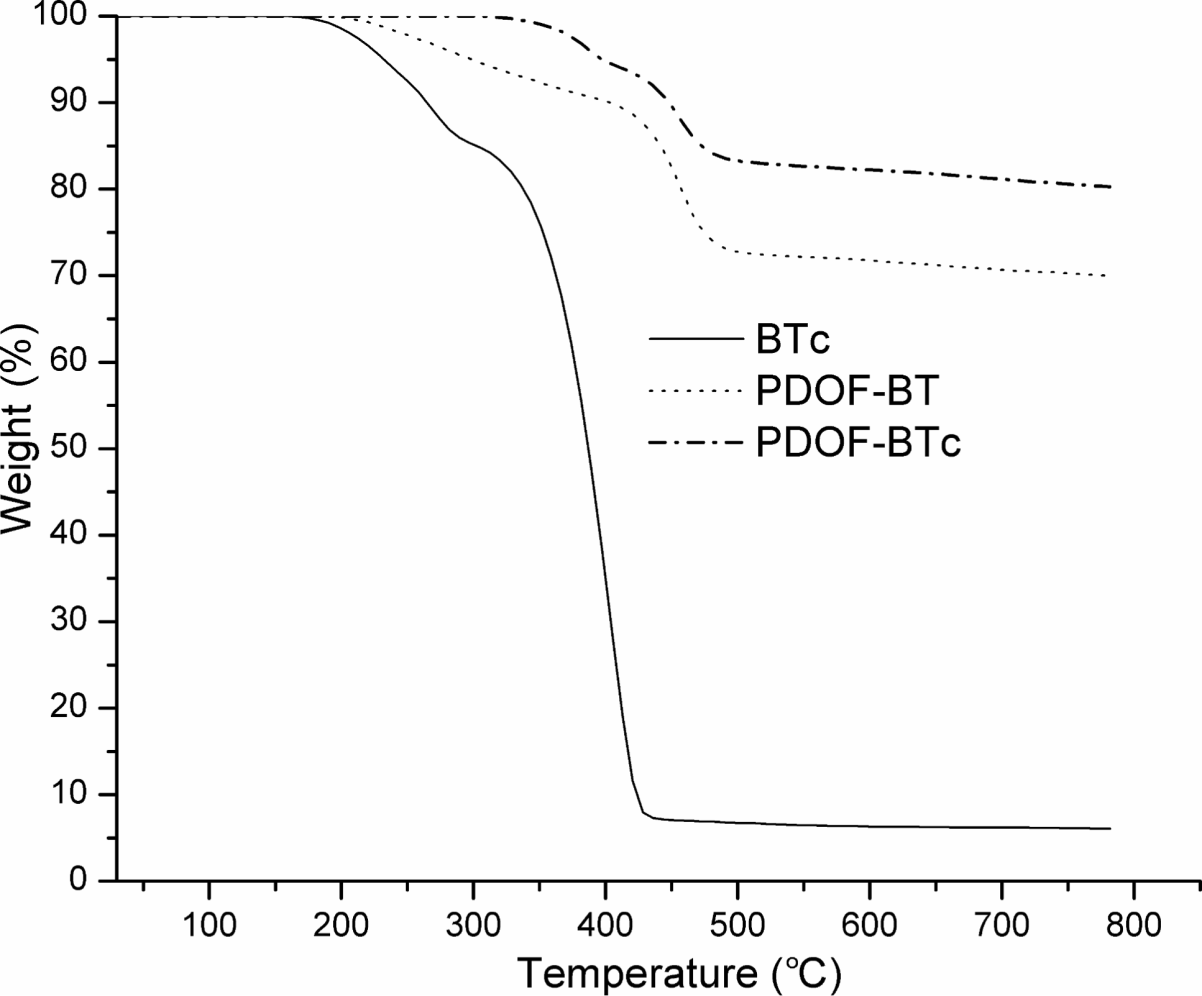
Thermogravimetric curves (TG) for **BTc**, **PDOF-BT**, and **PDOF-BTc** compounds.

The thermal stability of the inclusion-complex **BTc**, **PDOF-BT** and the **PDOF-BTc** copolymers was investigated by differential scanning calorimetry (DSC) and thermogravimetric analysis (TGA), as shown in Figure 5 and Figure 6, and the results are summarized in Table 2. The endothermic peak attributed to the melting temperature of **BT** (144 °C), as a result of a decomplexation process (17%) was present in the second heating scan of **BTc** (Figure 5). The presence of two broadening peaks accompanied by a shift to a lower melting temperature for **BTc** (133 °C, Δ*H* = 5.22 J/g) with a strong reduction of intensity could be attributed to a heating-favoured loosening of the crystal forces of **BT** included in the amorphous PS-βCD cavities.

**Table 2:** Thermal properties.

Sample	Step	*T*_onset_^a^	*T*_endset_^b^	*T*_peak_^c^	W%^d^	Residue^e^ %

**BTc**	I	196	287	271	15.49	63.18
	II	350	433	401	79.72	
**PDOF-BT**	I	212	330	264	9.78	69.07
	II	426	480	456	21.15	
**PDOF-BTc**	I	341	396	383	6.38	80.94
	II	431	480	456	12.68	

^a^The onset temperature of the degradation process. ^b^The temperature of complete degradation process. ^c^The maximum degradation temperature. ^d^The mass percentage loss recorded in each stage. ^e^The amount of residue at the end of degradation process.

On the cooling cycle (not shown) the exothermal sharp peak attributed to the crystallization process of **BTc** was found at lower temperature (108 °C, Δ*H* = 7.66 J/g)) compared with non-complexed **BT** (111 °C). DSC profiles of **PDOF-BTc** copolymer does not indicate *T*_g_ or melting temperature in the 30–200 °C interval, as can be seen in Figure 6. These findings indicated their amorphous nature and rigid chains.

The effect of PS-βCD on the thermal stability of the samples was further supported by TGA in a nitrogen atmosphere, which revealed the stage of the degradation process, shown in Figure 6 and Figure S4 in the Supporting Information File 1.

As one can see from Figure 6, **BTc**, **PDOF-BT** and **PDOF-BTc** samples present two thermal decomposition steps. For the **PDOF-BT** sample the decomposition process starts at 212 °C, while the degradation of its **PDOF-BTc** complex homologue begins at a higher temperature (341 °C). Thus, a stabilizing effect of the inclusion of the **BT** chain into the PS-βCD cavity for the first decomposition step is evidenced. The maximum degradation process of the samples is around 271 °C for **BTc**, 264 °C for **PDOF-BT** and 383 °C for **PDOF-BTc**, i.e., not much higher than the decomposition temperature of PS-βCD (380 °C). The TGA data revealed that the **PDOF-BTc** rotaxane copolymer is stable up to 300 °C. The improvement of the thermal stability provides an indication that the rotaxane formation increases the stability of the **PDOF-BT** macromolecular chains.

**Figure 7:**
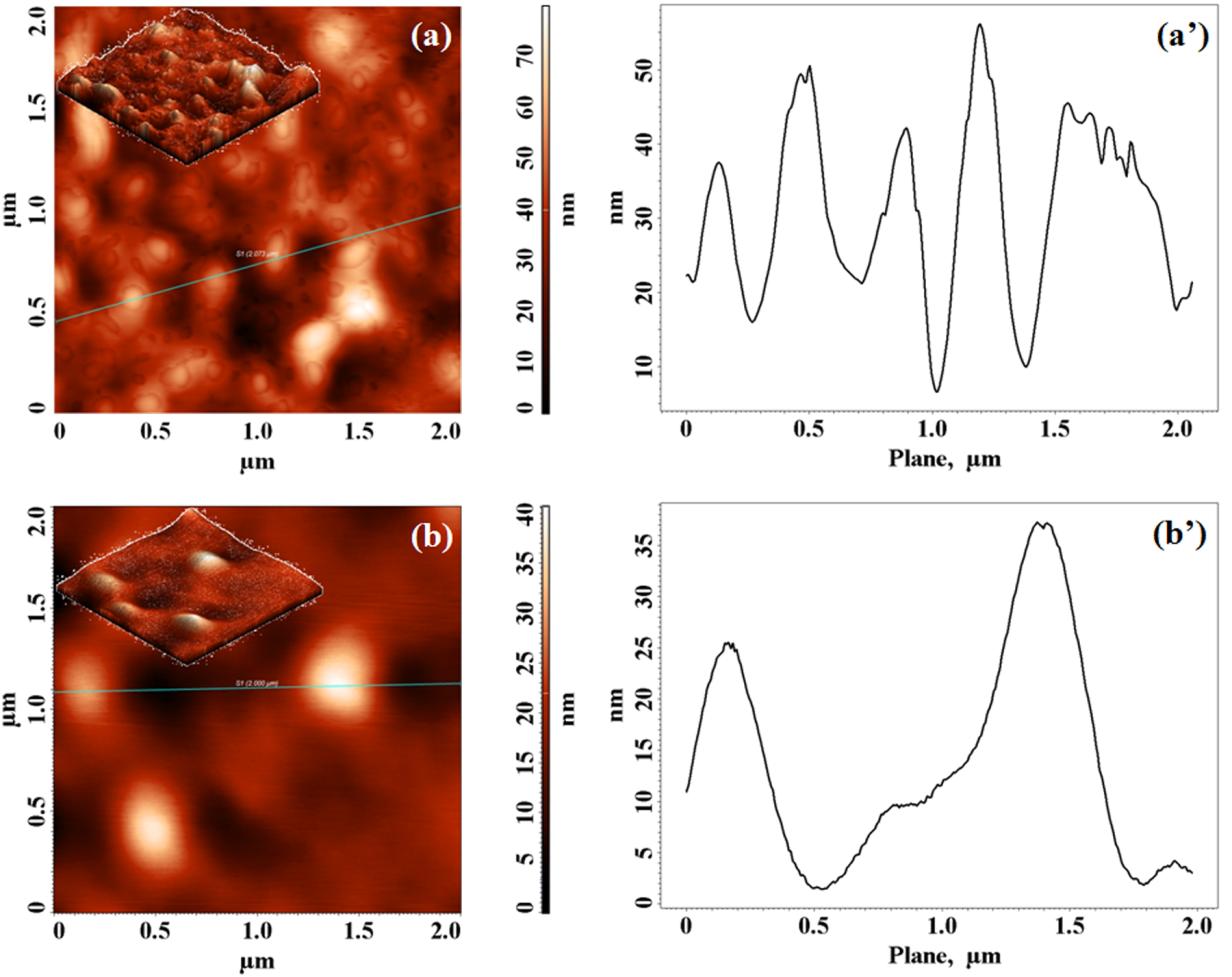
High-resolution tapping-mode AFM images and cross-section plots (along the solid line in the images) of the **PDOF-BT** (a, a’) and **PDOF-BTc** (b, b’).

The rotaxane copolymer has good solubility in common organic solvents, such as CHCl_3_, CH_2_Cl_2_, THF, and toluene, and allows the formation of homogeneous and transparent films. In order to analyse the influence on the surface morphological properties induced by using **3T** as an end-capping reagent instead of bromobenzene [12], AFM experiments were also performed. The 2D AFM images of the top surface of both copolymer films, within the same scan areas of 2 × 2 μm^2^ are shown in Figure 7a and Figure 7b (insets show the 3D images). They afforded the root-mean-square roughness of the formations (*S*_q_) and the average roughness (µ) as well as the average heights (*H*_a_), see Table 3.

**Table 3:** Roughness and grain parameters collected from 2 × 2 µm^2^ AFM images.

Material	*S*_q_^a^/nm	μ^b^/nm	*H*_a_^c^/nm

**PDOF-BT**	12.43	9.83	34.40
**PDOF-BTc**	5.29	3.70	17.34

^a^Root-mean-square roughness. ^b^Average roughness. ^c^Average heights.

The 3D AFM images of the complexed **PDOF-BTc** film (insets Figure 7b) show more favourable surface parameters in comparison to the noncomplexed copolymer (insets Figure 7a). **PDOF-BT** showed an agglomeration tendency and globular formations with *S*_q_ and µ values of 12.43 and 9.83 nm, whereas the **PDOF-BTc** sample showed a uniform and smooth surface, covered with individual small, spherically shaped formations, with smaller *S*_q_ and µ values of 5.29 and 3.70 nm. The average AFM heights of the **PDOF-BT** and **PDOF-BTc** decreased from 34.40 to 17.34 nm, respectively, as shown in Figure 7a’ and Figure 7b’ and documented in Table 3.

The trend toward a more uniform and smoother surface, already observed for rotaxane copolymers [12,23,27,31], continues for the **PDOF-BTc** polymer rotaxane, where a decrease of the *S*_q_ and the value of the *H*_a_ parameter (the first moment of the height distribution) indicated that the rotaxane architecture changed the morphology of **PDOF-BT** copolymer. These results could provide microscopic evidence for reduced interchain interactions, which allow the polyrotaxane chains to pack densely. A better quality of the film, with higher uniformity, flatness, and better mechanical properties, is always desirable for optoelectronic applications.

The optical properties of the **PDOF-BT** and **PDOF-BTc** compounds (not shown) recorded in CHCl_3_ solution, as well as in thin film, upon excitation at the maximum absorption value (λ_max_ = 380 nm) indicate no significant difference in the electronic absorption and emission spectra of both copolymers, consistent with previously reported results [12]. The similarity between the absorption and fluorescence excitation spectra shows that major structural changes have not occurred, and that emissions arose from the compounds and not from the thienyl end-capping, as has been previously reported for PFO with perylene derivative as end-units [35].

## Conclusion

The present study confirms that PS-βCD can be used as a host macrocycle molecule in the synthesis of a main-chain polyrotaxane with alternating fluorene-bithiophene moieties. Lower values of number-average molecular weight (*M*_n_) of the rotaxane copolymer suggested that the condensation reaction is subjected to steric effects of the bulkier silylated groups from PS-βCD, thus requiring a longer reaction time. We have demonstrated that PS-βCD covered bithiophene-fluorene copolymer has beneficial effects on the solubility in nonpolar solvents, and on the thermal properties and surface characteristics.

## Experimental

### Materials and methods

PS-βCD was obtained by the silylation of native βCD with 1-trimethylsilylimidazole [36]. 9,9-dioctylfluorene-2,7-bis(trimethylene borate) (**DOF**, 97%), tetrakis(triphenylphosphane)palladium (99%), and 5,5^'^-dibromo-2,2'-bithiophene (**BT**) were purchased from Aldrich and used as received; 3-bromothiophene (**3BT**, 97%) was purchased from Lancaster. All solvents were analytical grade and used without further purification.

^1^H NMR spectra were recorded in CDCl_3_ on a Bruker Advance 400 MHz instrument. FTIR analyses of the powder polymers were performed in a Specord Carl Zeiss Jena FTIR spectrophotometer. The molecular weights of copolymers were determined by gel permeation chromatography (GPC) in CH_2_Cl_2_ by using a Water Associates 440 instrument and polystyrene calibrating standards. Differential scanning calorimetry (DSC) was performed with a Mettler Toledo DSC-12E calorimeter with two repeated heating-cooling cycles at a heating rate of 10 °C·min^−1^ under a N_2_ atmosphere. Thermogravimetric analysis (TGA) was performed under constant nitrogen flow (20 mL·min^−1^) with a heating rate of 15 °C·min^−1^ by using a Mettler Toledo TGA/SDTA 851e balance. The heating scans were performed with 1.5 to 3 mg of the sample in a temperature range 25–800 °C. Absorption spectra were measured on a Specord 200 spectrophotometer in CHCl_3_ solution and on thin films. Fluorescence spectra were obtained with a Perkin Elmer LS55 luminescence spectrophotometer. The surface images were obtained with a Solver PRO-M scanning probe microscope (NT-MDT, Zelenograd, Moscow, Russia), in atomic force microscopy (AFM) configuration. The scan area was 2 × 2 µm^2^. Rectangular silicon cantilevers NSG10 (NT-MDT, Russia) with tips of high aspect ratio were used. All images were acquired in air, at room temperature (23 °C), in tapping mode at a scanning frequency of 1.56 Hz. The AFM image processing and the calculation of the surface texture parameters were realized by Nova v.1.26.0.1443 for Solver Software, NT-MDT Russia. Films of copolymers were prepared by spin coating from CH_2_Cl_2_ solutions at 3000 rpm for 60 s on a WS-400B-6NPP-Lite Single Wafer Spin Processor (Laurel Technologies Corporation, USA).

### Synthesis of the **BTc** inclusion complex

**BT** (0.124 g, 0.4 mmol) was added to the solution of PS-βCD (2.58 g, 0.96 mmol) in acetone (15 mL) and the mixture was vigorously stirred for 24 h. The precipitate was filtered, washed thoroughly twice with 5 mL of petroleum ether and 5 mL of acetone, and finally dried under vacuum at 60 °C for 24 h to yield 0.607 g of **BTc** as a light-yellow solid (42.9% yield). ^1^H NMR **BTc** (CDCl_3_): 6.97–6.96 (d, 2H), 6.85–6.86 (d, 2H), 5.12–4.95 (m, 7H, H^1^), 4.93–4.60 (m, OH^2+3^), 3.97–3.35 (m, 42H, H^2–6^), 0.19–0.09 (m, 63, Si–CH_3_).

### Synthesis of the **PDOF-BTc** copolymer

To a three-necked flask, 0.602 g (0.202 mmol) of **BTc** and 0.112 g (0.2 mmol) of **DOF** was added. The flask was equipped with a condenser, evacuated, and filled with argon several times to remove traces of air. Degassed toluene (6 mL) was added as solvent into the flask and subsequently 4.8 mg (0.42 × 10^−2^ mmol) of (Ph_3_P)_4_Pd(0), dissolved in 5 mL of degassed toluene, and 2 mL of 5 M Na_2_CO_3_ solution were added. The mixture was vigorously stirred in the dark under an argon atmosphere for 72 h at 85–87 °C. An excess of 0.0113 g (0.02 mmol) of **DOF** dissolved in 3 mL of toluene was then added and the reaction was continued for 12 h in order to obtain the macromolecular chains terminated with borate units. Finally, 0.2 μL of **3BT** was added as the end-capper of the copolymer chain, and the reaction was continued overnight. After cooling, the mixture was poured into the stirred mixture of methanol and deionised water (10:1). The fibrous solid obtained by filtration was solubilised in 20 mL of toluene, washed with water three times to completely remove the alkali solution, dried over anhydrous MgSO_4_, and concentrated by vacuum evaporation of the solvents. The residue was dissolved in a minimum volume of CHCl_3_ (10 mL) and poured into 10 times the volume of stirred methanol, and then filtered, thoroughly washed with 5 mL of acetone, and dried under reduced pressure at 60 °C. **PDOF-BTc** was obtained in 0.125 g (approximately 23.0% yield) as a light-orange solid. After drying, the solid was immersed in petroleum ether, vortex stirred for 15 min, and then filtered. The precipitate was filtered and dried under vacuum. The soluble part in petroleum ether was concentrated by vacuum evaporation. Both fractions of **PDOF-BTc** were found to be also soluble in THF, CH_2_Cl_2_, CHCl_3_ and toluene. FTIR (KBr, cm^−1^): 3447, 2957, 2923, 1608, 1415, 1364, 1251, 1144, 1096, 1049, 966, 885, 841, 748, 687, 542, 475, 434. ^1^H NMR (CDCl_3_): 7.67–7.57 (m, 6 H), 7.33–7.24 (m, 4H), 5.13–4.95 (m, 7H, H^1^), 4.95–4.79 (m, OH^2+3^), 3.96–3.53 (m, 42H, H^2–6^), 2.04 (s, 4H), 1.25–0.88 (m, 24H), 0.74 (d, 6H), 0.18–0.07 (m, 63, Si–CH_3_).

### Synthesis of the **PDOF-BT** copolymer

**PDOF-BT** was synthesized under similar experimental conditions as those described for **PDOF-BT**, except that **BT** was used instead of **BTc**. The copolymer was obtained as a brownish solid in 78% yield. The **PDOF-BT** sample was insoluble in petroleum ether. FTIR (KBr, cm^−1^): 3428, 3065, 2923, 2851, 1719, 1606, 1465, 1261, 1067, 880, 817, 721, 475, 434. ^1^H NMR (CDCl_3_): 7.68–7.58 (m, 6 H), 7.34–7.24 (m, 4H), 2.05 (m, 4H), 1.26–1.10 (m, 24H), 0.91–0.80 (d, 6H).

## Supporting Information

File 1 Gel permeation chromatography and derivative thermogravimetric curves.
