# Supplementary material for: Synthesis and characterization of low-molecular-weight π-conjugated polymers covered by persilylated β-cyclodextrin
Source: Beilstein J Org Chem. 2012 Sep 11;8:1505–14. doi: 10.3762/bjoc.8.170 (PMC3458775; doi:10.3762/bjoc.8.170)
Supplement: File 1 — Gel permeation chromatography and derivative thermogravimetric curves. [file Beilstein_J_Org_Chem-08-1505-s001.pdf]

Supporting Information

for

Synthesis and characterization of low-molecular-weight π-conjugated polymers covered by persilylated β-cyclodextrin

Aurica Farcas*, Ana-Maria Resmerita, Andreea Stefanache, Mihaela Balan and Valeria Harabagiu

Address: Inorganic Polymers, ‘‘Petru Poni’’ Institute of Macromolecular Chemistry, Grigore Ghica Voda Alley, 700487-Iasi, Romania

Email: Aurica Farcas - afarcas@icmpp.ro

* Corresponding author

Gel permeation chromatography and derivative thermogravimetric curves

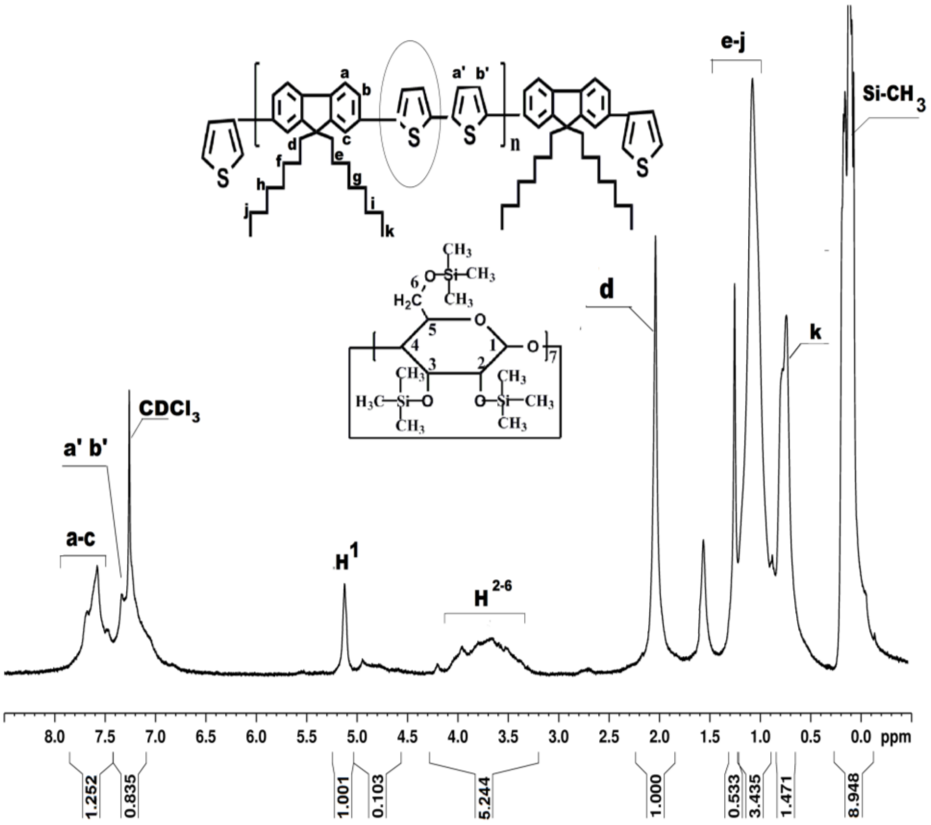

Figure S1: ^{1}H NMR spectrum of **PDOF-BTc** rotaxane copolymer (soluble part in petroleum ether).

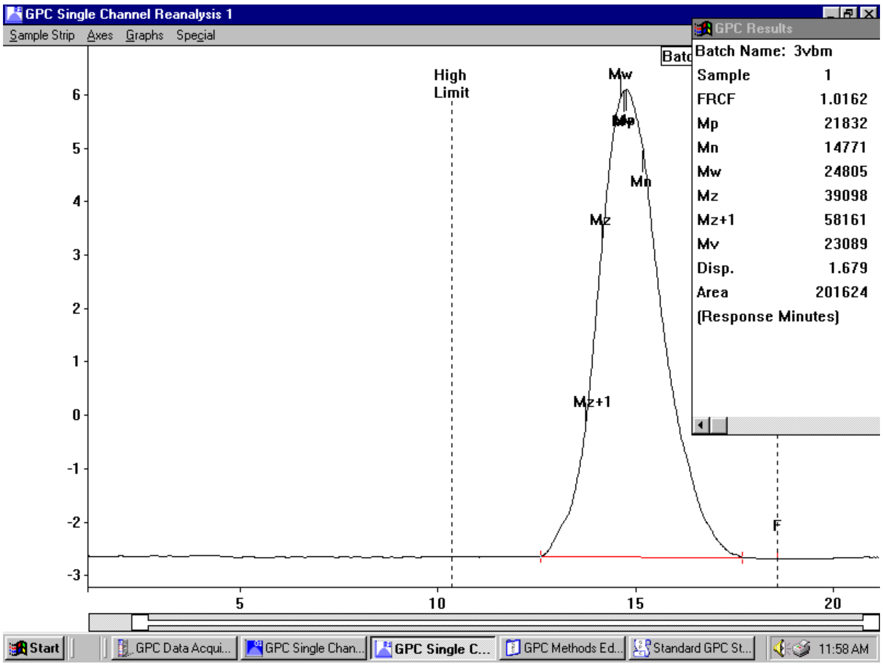

Figure S2: Gel permeation chromatography (GPC) of **PDOF-BTc** rotaxane copolymer.

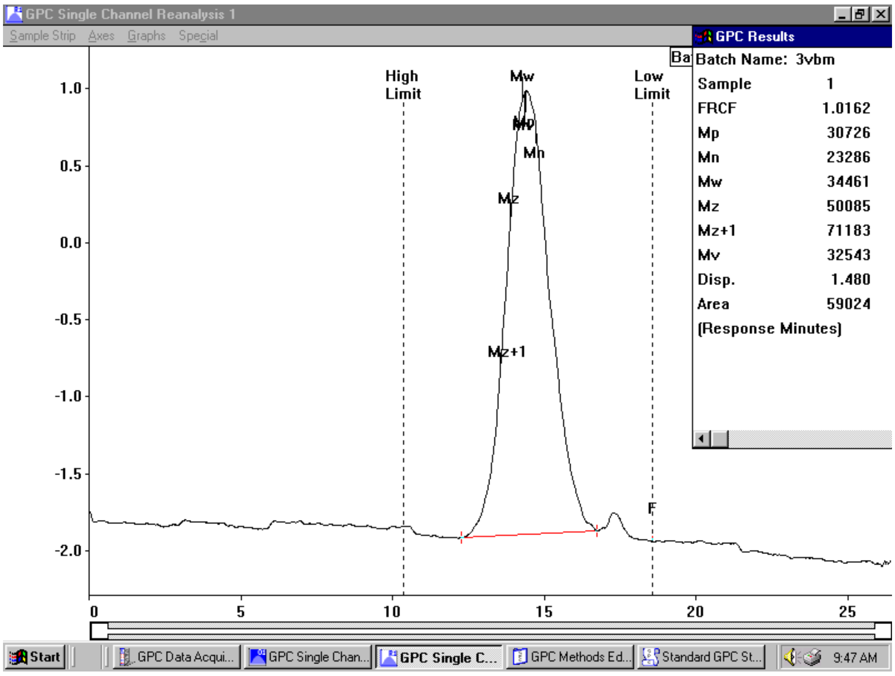

Figure S3: GPC of **PDOF-BT** non-rotaxane copolymer.

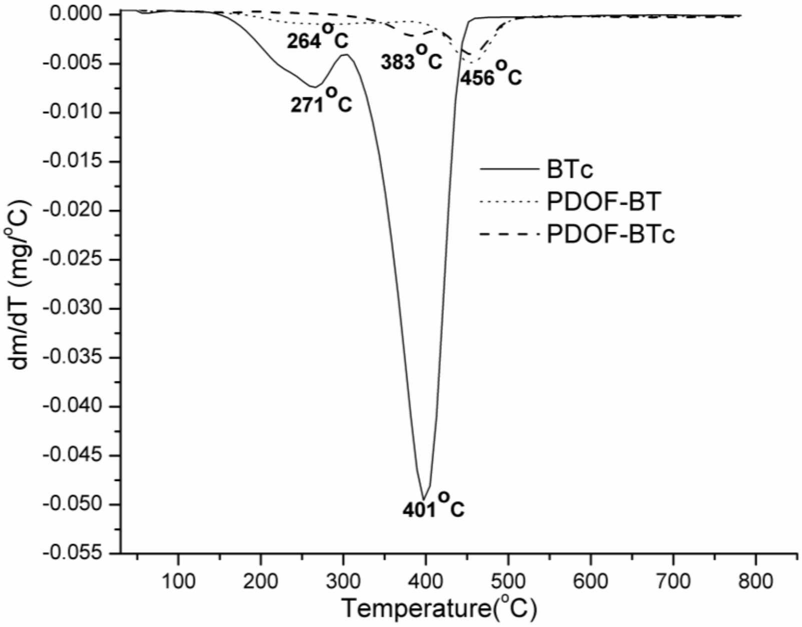

Figure S4: Derivative thermogravimetric curves (DTG) of **BTc**, **PDOF-BT** and **PDOF-BTc** samples.
